# Prevalence and risk factors of disabilities among Egyptian preschool children: a community-based population study

**DOI:** 10.1186/s12888-023-05171-3

**Published:** 2023-09-21

**Authors:** Ammal M. Metwally, Ahmed Aboulghate, Ghada A. Elshaarawy, Ali M. Abdallah, Ehab R. Abdel Raouf, Ebtissam M. Salah El-Din, Zeinab Khadr, Mostafa M. El-Saied, Mona A. Elabd, Maysa S. Nassar, Marwa W. Abouelnaga, Engy A. Ashaat, Mohamed M. El-Sonbaty, Hala Y. Badawy, Eman M. Dewdar, Somia I. Salama, Mohamed Abdelrahman, Aida M. Abdelmohsen, Sherif E. Eldeeb, Maie M. Naga, Nada H. Elshamy, Fatma A. Shaaban, Amira S. ElRifay

**Affiliations:** 1 Community Medicine Research Department, Medical Research and Clinical Studies Institute, National Research Centre (ID: 60014618), P.O. 12622, Giza, Dokki Egypt; 2 Quantitative Methods Department, Aswan University, Aswan, Egypt; 3 Child With Special Needs Department, Medical Research and Clinical Studies Institute, National Research Centre (Affiliation ID: 60014618), Cairo, Dokki Egypt; 4 Child Health Department, Medical Research and Clinical Studies Institute, National Research Centre (Affiliation ID: 60014618), Cairo, Dokki Egypt; 5 Department of Statistics, Faculty of Economics and Political Science, Cairo University, Cairo, Egypt; 6 The Social Research Center, American University in Cairo, Cairo, Egypt; 7 Clinical Genetics Department, Human Genetics and Genome Research Institute, National Research Centre (Affiliation ID: 60014618), Cairo, Dokki Egypt; 8 Prevention of Disability General Directorate, Ministry of Health and Population, Cairo, Egypt

**Keywords:** Disability, Preschool children, Vision, Hearing, Speech, Communication, Mobility, Intellectual impairment, Seizures

## Abstract

**Background:**

Child disability has significant implications on their well-being and healthcare systems. Aim: This survey aimed to assess the magnitude of seven types of disability among Egyptian children aged 1 < 6 years and their socio-demographic, epidemiological, and perinatal predictors.

**Methods:**

A national population-based cross-sectional household survey targeting 21,316 children from eight governorates was conducted. The screening questionnaire was derived from the WHO ten-question survey tool validated for identifying seven disability categories.

**Results:**

The percentage of children with at least one disability was 8.1% as follows: speech/communication (4.4%), Mobility/physical (2.5%), Seizures (2.2%), Comprehension (1.7%), Intellectual impairment (1.4%), Visual (0.3%) and Hearing (0.2%). Age was not found to affect the odds of disability except for visual disability (significantly increased with age (AOR = 1.4, 95% CI:1.1–1.7). Male sex also increased the odds of all disabilities except visual, hearing, and seizures. Convulsions after birth significantly increased the odds of disability as follows: hearing (AOR = 8.1, 95% CI: 2.2–30.5), intellectual impairment (AOR = 4.2, 95% CI: 2.5–6.9), and mobility/physical (AOR = 3.4, 95% CI: 2.3–5.0). Preterm delivery and being kept in an incubator for more than two days after birth increased the odds for visual disability (AOR = 3.7, 95% CI: 1.1–12.1 & AOR = 3.7, 95% CI: 1.7–7.9 respectively). Cyanosis increased the odds of seizures (AOR = 4.7, 95% CI: 2.2–10.3). Low birth weight also increased the odds for all disability domains except for visual and hearing. Maternal health problems during pregnancy increased the odds for all types of disability except hearing and seizures. Higher paternal education decreased the odds for all disabilities by at least 30% except for vision and hearing.

**Conclusion:**

The study found a high prevalence of disability among Egyptian children aged 1–6 years. It identified a number of modifiable risk factors for disability. The practice of early screening for disability is encouraged to provide early interventions when needed.

**Supplementary Information:**

The online version contains supplementary material available at 10.1186/s12888-023-05171-3.

## Background

The UN Convention on the Rights of Persons with Disabilities (UNCRPD) defined children with a disability as “those who have long-term mental, physical, or sensory or intellectual impairments which impact their completely effective participation in society and participation with the others” [1]. Oulanyah and colleagues estimated the number of children with developmental disabilities among children younger than 5 years in North Africa and the Middle East by 6.53 million [2] The investigated disabilities included visual and hearing impairments, seizures, and intellectual disabilities. Egypt has ranked as one of the top ten countries with these developmental disabilities apart from hearing impairments [2]. Studies have shown that children with disabilities have lower chances of entering school, lower attendance rates, lower grades, lower chances to higher levels of education, and lower quality of educational experience compared to their non-disabled peers [3–5]. In addition, the family challenges faced by the caregivers, especially mothers, were described as a feeling of heavy responsibility, constant worries about their children's needs, and having to help their children maintain normal community connections [6, 7]. Mental and mixed disabilities were found to be associated with an increase in the overall burden on caregivers [8]. This explains why the quality of life was significantly worse for caregivers who care for patients with both physical and mental diseases [9].

The systematic analysis of the Global Burden of Disease showed an increase in all developmental disabilities in the Middle East between 1990 and 2016 despite a decline observed globally in the same period [2]. There is a paucity of studies addressing the prevalence of disabilities among Egyptian preschool children. Previous studies showed high variability in prevalence (0.7–8.8%) and were conducted on samples that geographically do not represent the whole country [10, 11]. The high variability could at least in part be attributed to the difference in the method of data collection, the age of children at which screening was done, types of disabilities investigated, and societal attitudes (e.g. Tending to hide a disability in public). The most prevalent types of disability reported were visual, speech, and hearing disabilities (4.5%, 2.1%, and 1.9%, respectively) [11, 12].

The major causes of disability in Egypt reported were congenital abnormalities, followed by injuries/accidents, epidemics, other chronic diseases, and birth-related conditions [11]. Many of the reported disabilities are preventable with adequate public health efforts [13], like hearing impairment resulting from chronic untreated ear infections [14, 15]. Also, multiple intellectual disabilities are caused by birth injuries due to the unavailability of perinatal care services [16–18].

In this study, we aimed to quantify the national prevalence of disabilities among Egyptian children aged 1–6 years through a household survey. We also aim to identify their risk factors out of the sociodemographic, epidemiological characteristics, and perinatal medical history. The results will help guide the screening for at-risk children and help in the early diagnosis and proper management of disabilities.

## Methods

### Study type

This study was a cross-sectional national prevalence survey, conducted on a house-to-house basis over a period of 24 months starting from December 2017 till December 2019**.**

### Target group and subjects inclusion criteria

The study focused on children aged 1year to less than 6 years at the visited houses and who were belonging to the mentioned governorates, locality and sociodemographic status provided in the supplementary file (S Table 1). Children aged 1–6 years whether experienced normal milestones for their ages or who met the definition of disabilities [19, 20], were included in the current study.

### Sampling frame and cluster preparation

**Fig. 1:**
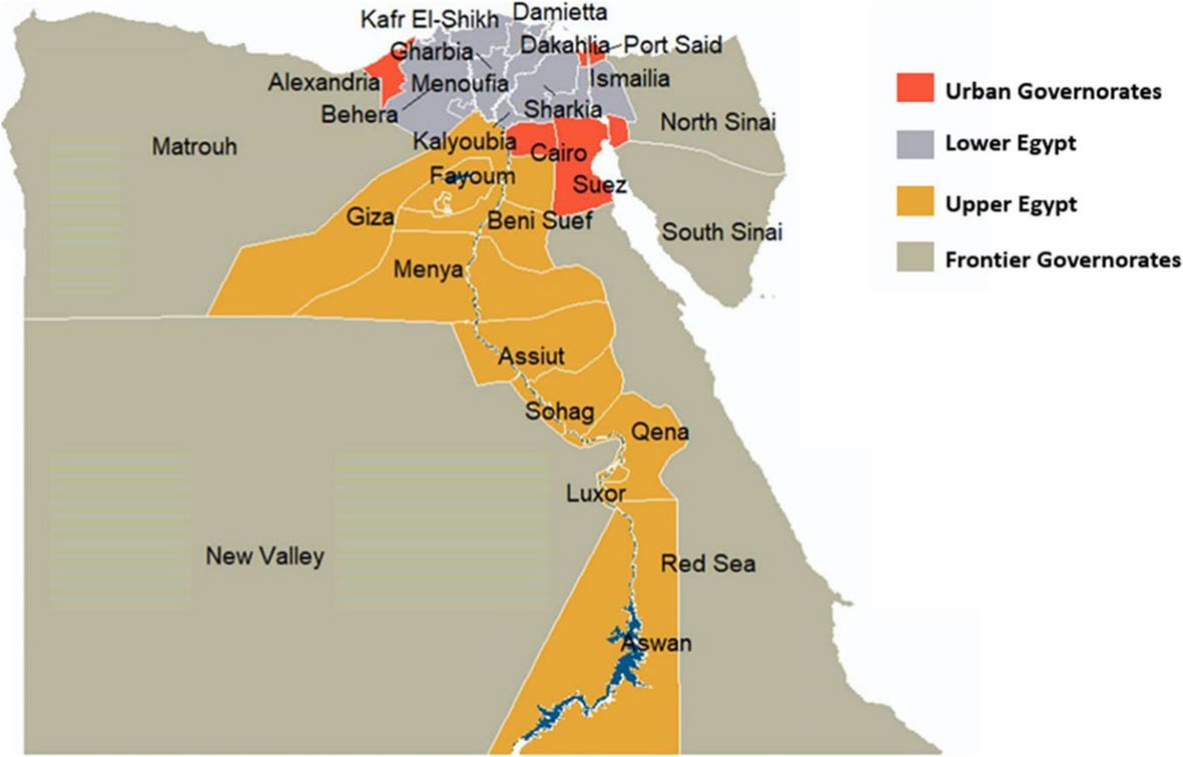
Map of the 27 Egypt's governorates distributed within the four geographic regions (adapted using data from the Humanitarian Data Exchange under the CC BY-IGO license [22]

The survey sample was nationally representative with multistage sampling technique and three sampling frames as three stages. The first sampling frame used was the comprehensive list of the 27 governorates of Egypt, according to the enumeration census from the Central Agency for Public Mobilization and Statistics (CAPMAS) [21] within each of the four main geographic administrative regions of Egypt as shown in Fig. 1.

In the first stage, a representative sample of 8 governorates was randomly selected to represent the main geographic areas in Egypt according to governorates in 2017 census which revealed that population percentage in urban governorates reached 17.1% of the total population, 43% in Lower Egypt versus 38% in Upper Egypt, while Frontier governorates only represented 1.7% of the total population in the same year. Accordingly, the following governorates were selected: one urban governorate (Cairo), 3 governorates of Upper Egypt (Fayoum, Assuit, and Aswan), 3 governorates of Lower Egypt (Damietta, Dakahlia and Gharbia) and one Frontier governorates (Marsa Matrouh).

Egyptian governorates are further subdivided into urban cities (Kism) and rural local village unit (Markaz). Egypt contains 177 cities and 162 local village units [17].

In the second stage, a representative sample of cities and local units was selected from each governorate. In this step, the design of the sample took in consideration the differences in human development within each governorate. Using the human development index produced by the UNFPA (2003), [23–25] each governorate was divided into three categories according to their human development scores, namely low, medium and high. From each category, one city and one local unit were selected from each governorate.

In the third stage, all Shiakha and villages of each of the randomly selected Kism and Markaz were listed as clusters with random selection of one Shiakha and one village per social category of each of the chosen governorate. The study finally included 45-blocks of Shiaka and villages (within 24 Kism in urban areas and 21 Markaz in rural areas respectively) to ensure heterogeneity of the data collected (S Table 1). In this stage, households in the selected city and village blocks were screened.

The sample was proportional to size for large governorates. For governorates with relatively small populations, an arbitrary sample size was assigned with adjusting weights during the analysis of the data. The weights were developed according to the total number of children aged 1—< 6 years in each governorate [26].

### Sample size calculation

Sample size calculation was based on the expected prevalence of disability ranging from 1.9% for hearing to 4.5% for visual disability as suggested by previous studies [11]. The 1996 and 2006 Egyptian censuses reported disability rates of 0.5% and 0.6% among all age groups [27].

The prevalence of any disabilities was 4.8% among youth [12]. The level of accuracy was set at 0.0049 (margin of error), confidence limit of 95%. The approximate average number of children in the age range 1–6 years within each family is 2 [28, 29]. According to the basis mentioned in the study for sample size calculation, the expected sample size after the addition of 10% expected non-response was 21,906. The actual targeted children were 21,392 with 2.3% of losses. Completed questionnaires were reported in 21,316 children out of the targeted 21,392 with 0.4% loss rate. The expected and the actual targeted numbers of children were mentioned according to the governorates, locality and sociodemographic status for children aged 1–6 years (S Table 1).

We used implicit stratification which is a form of geographic stratification that is used together with probability proportionate to size (pps). Probability proportionate to size was the used technique of selecting sample areas to be proportional to their population.

### Screening questionnaire

The developed and used assessment questionnaire for disability utilized the validated WHO Ten questions screening tool (TQS) [30, 31] and the questions designed by the United Nations (UN) Washington Group on Disability Statistics (WG) to make meaningful comparisons of disability prevalence between nations for disability detection, which were validated for children aged up to 17 years.

The advantage of using the TQS lies in that: The TQS is looking specifically at activity limitations and participation restrictions (ICF framework). It has the advantage of focusing on universal abilities and is considered to be cross-culturally comparable. It is shown to be equally valid for girls & boys (not to be gender biased). The sensitivity of TQS in detecting disability was 100% with a high positive predictive value [32]. With the aid of this tool functional difficulties in children across several domains were assessed to identify children who are at greater risk of experiencing limited participation in an unaccommodating environment, including vision, hearing, speech, mobility, communication/comprehension, learning, seizures, and intellectual disabilities.

The questions used to identify and investigate these disability forms by this tool were included as the first part of the household questionnaire: 1) difficulty seeing (in the daytime or night, even if wearing glasses, 2) difficulty hearing, even if using a hearing aid, 3) comprehension (unable to understand orders), 4) movement (weakness or stiffness in the arm(s)/leg(s) with difficulty walking or climbing stairs, 5) seizure (have fits, rigid or lose consciousness), 6) learning (unable to do something like other children his/her age), 7) speech (no speech), 8) communication (unclear speech) and 9) intellectual impairment (appeared mentally backward/dull or slow). The categorization of disabilities was based on the WHO, International Classification of Functioning, Disability, and Health, Version for Children and Youth [19, 20], to include seven categories for which learning, and comprehension were included in the same category, Speech and communication were also included in another category. Developmental millstones (for any delay in sitting, standing, or walking), were not included or discussed, in this study being verified by other more validated tools with published data [33].

Limitation of TQS: Precision of caregiver awareness of child’s development and behavior relative to own cultural norms remains controversial. Some studies have shown a high level of agreement between caregiver awareness and professional identification [34, 35]. The type and severity of disability may affect the level of agreement. Caregivers’ concerns about language, emotional/behavioral, and motor developmental delays are adequate indicators of children’s developmental status. However, caregivers usually had difficulties to identify the signs of potential cognitive or global developmental problems correctly if the child was not severely [34, 36].

The second part of the household questionnaire covered information on housing characteristics e.g., the number of rooms, the source of water, and ownership of a variety of consumer goods. The households’ characteristics age, sex, marital status, educational attainment, work status, and relationship to the household head) and possessions were used to develop a wealth index for the interviewed households to define the standards of living.

The third part of the questionnaire included information about perinatal medical history of the mother and the target child.

Arabic translation and back translation to of the screening questionnaires and pretesting with 80 caregivers (10 per governorate) were conducted before the fieldwork to ensure the understandability of disabling conditions in the Arabic culture. A pre-tested questionnaire was then adjusted according to the pretesting results. Once the questionnaire was in its final form printing, the data entry coding took place.

### Data collection strategy, office editing, coding and data processing

Data collection was implemented by professional field surveyors who were 64 social workers. Before the survey implementation, a preparation phase was done to ensure quality control, contacting local authorities. Condensed training sessions about how to conduct the WHO TQS questionnaire in a standardized way were done. A completed inter-rater reliability test for the WHO TQS was downdone; all achieved above 90%. The survey was then conducted under the supervision of collaborative team from Cairo Demographic Centre (CDC) with the professional team members from the National Research Centre of Egypt (NRC). The implementation of the screening was carried out at the household level. The data collectors visited the assigned numbers of homes to run the study (S Table 1). The parents of the children were briefed on the objectives of the study and how it would be administered. A written consent from the parents/caregivers of children aged 1- < 6 years to participate in the study was taken. The 10 questions WHO screening and verified Arabic questionnaire was directed to parents/caregivers through face-to-face interviews.

Recognized children with any form of the studied disabilities according to WHO TQS were confirmed by specialized physicians in the health care centers of the Ministry of Health and Population (MOHP) and NRC to ascertain the results of the screening phase. The professional team members of the NRC ensured that recognized children with any detected form of disability or delay who agreed to be managed in the rehabilitation programs of the MOHP were enrolled in these centres for free.

Office editors reviewed the questionnaires for internal consistency and completeness. Coding of questionnaires used was conducted at the office prior to the data entry. One senior staff member and 3 office editors were recruited for this purpose. A CSPro database program was developed by a software developer and was used for data entry purposes. Around 6 data entry personnel were recruited and trained for this purpose. Five percent of the questionnaires were re-entered for verification.

### Quality control

The quality control of data was performed through the following steps: Selecting and training qualified field staff, Field editing by field editors and supervisors, Field checking and re-interviewing by quality control personnel and general supervisors, Office editing and Re-entry of 5% of questionnaires.

### Statistical analysis

Frequencies and proportions as well as means ± standard deviation (SD) were used to describe categorical and continuous variables, respectively. Comparisons between groups were done using odds ratios (OR) and 95% confidence intervals (CI) were calculated in comparison between children having disabilities and healthy children. Factors that were found to be statistically significant in the univariate logistic regression analysis were subjected to multivariate logistic regression (Enter Wald) for adjusting and controlling the effect of confounding variables to determine the predictors (risk factors for the studied disability types and those without disability) based on the values of the independent variables [30]. Results were presented in terms of crude odds ratio (COR) and adjusted odds ratio (AOR) in a univariate and multivariate analysis respectively. Variables with *p*-values of < 0.2 during the bivariable analysis were fitted to the multivariable logistic regression analysis. A significant association is considered if the 95% CI does not include the value 1.0. Finally, a cutoff *p*-value of less than 0.05 is used to declare statistical significance. All statistical analyses were carried out using Statistical Package for Social Sciences (SPSS) software version 22.0 software (IBM SPSS Statistics for Windows, Version 22.0. Armonk, NY: IBM Corp.).

## Results

**Fig. 2:**
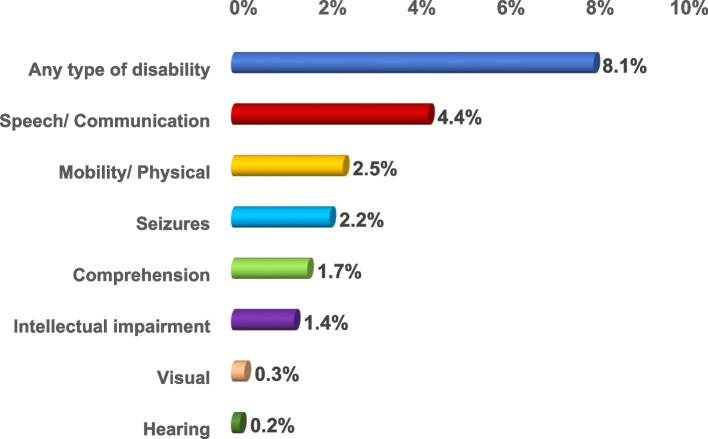
Prevalence of the types of disabilities out of the 21,316 surveyed Egyptian children aged 1– < 6 year

The prevalence of disabilities among children aged 1 – < 6 years was 8.1%. The prevalence of disability types was as follows: speech/ communication (4.4%), mobility/ physical (2.5%), seizures (2.2%), comprehension (1.7%), intellectual impairment (1.4%), visual (0.3%) and the least was for hearing (0.2%) (Fig. 2).

The total number of surveyed children aged 1 – < 6 years was 21,316 (Table 1). Boys represented 52% of the whole sample versus 48% of girls. The surveyed children were insignificantly higher among the rural than the urban localities, equally distributed among social classes. Regarding the age distribution, children aged 3- < 6 years represented the largest portion 60.7%.

**Table 1:** Characteristics of the study population

Socio-demographic characteristics	Surveyed children (21,316)	
	***N***	**Column%**
**Locality (Urban/ Rural)**		
Urban	9707	45.5
Rural	11,609	54.5
**Social class**		
Low	6924	32.5
Middle	7093	33.3
High	7299	34.2
**Geographical Distribution**		
Cities	3264	15.3
Lower Egypt	7921	37.2
Upper Egypt	7705	36.1
Frontier	2426	11.4
**Sex**		
Boys	11,076	52.0
Girls	10,240	48.0
**Age category**		
Mean child age (year) ± SD	3.5 ± 1.5	
1- < 3 years	8378	39.3
3- < 6 years	12,938	60.7
**Current mother age**		
Mean age (year) ± SD	30.1 ± 5.9	
**Mother age at giving birth**		
Mean age(year) ± SD	26.6 ± 5.8	
< 18	938	4.4
18 to < 35	18,376	86.2
≥ 35	1898	8.9
**Mother Education**		
Illiterate/ Read & write/ Primary/ Prep	8150	38.2
High School & technical/ above intermediate	9432	44.2
University or higher	3622	17.0
**Father Education**		
Illiterate/ Read & write/ Primary/ Prep	7129	33.4
High School & technical / Above intermediate	9867	46.3
University or higher	3449	16.2
**Mother work**		
Work (paid-unpaid-his own-employer)	2976	14.0
Unemployed	18,225	85.5
**Mean of HH members ± SD**	4.8 ± 1.3	
**Presence of mothers or fathers**		
No father in the HH	862	4.0
No mother in the HH	109	0.5
**Twin child**	975	4.6
**Disabled mothers**^a^	165	0.8
**Disabled fathers**^a^	319	1.5
**Perinatal problems**		
Premature children (< 37 weeks gestation)	229	1.1
Low birth weight (< 2500 mg)	1077	5.1
Children suffer from jaundice after birth	6180	29.0
Children suffer from bluish discoloration after birth (Cyanosis)	313	1.5
Children suffer from any convulsions	355	1.7
Children kept in an incubator for more than two days	1770	8.3
Mothers have any health problem during pregnancy^b^	1500	7.0
Difficult labor^c^	3435	16.1

^a^Disabled mothers or fathers: physically or mentally disabled; Hearing, Vision, Mental, Movement, Speech [19, 20]

^b^Mothers having any pregnancy complications such as iron deficiency anemia, gestational diabetes, hypertension, infection, anxiety or depression [37]

^c^Difficult labor refers to prolongation in the duration of labor, typically in the first stage of labor. It can be an important contributor to maternal and perinatal mortality and morbidity if it remains unrecognized or untreated [38]

Most of the mothers' age at giving birth was in the age range 18—< 35 years (86.2%). Most of mothers and fathers had high school or technical and above intermediate education (44.2% and 46.3% respectively). Most of the mothers were housewives and unemployed. Houses without mothers were 0.5% versus 4.0% headed by mothers without fathers. Mothers and fathers with apparent disabilities were 0.8% and 1.5% respectively. Twin children represent 4.6% of the surveyed children. The presence of neonatal jaundice was the most prevalent perinatal problem (29%).

Table 2 shows the odds of having handicapping disabilities. Concerning the sociodemographic factors, the odds of the presence of children with at least one disability was significantly one and a half higher in cities than in frontier, upper and lower Egypt governorates with a prevalence of 11.0%, 6.9%, 7.7%, and 7.7% respectively. The odds were 1.3 times higher among urban than rural communities (prevalence 9.2% vs. 7.2%) and among middle and low than high class (prevalence 8.7% and 8.3% respectively vs. 7.3%).

**Table 2:** Sociodemographic and epidemiological characteristics of the studied population according to disabilities

**Socio-demographic and epidemiological characteristics**	**Children with at least one disability (total children with disabilities)** ***>N*** **= 1727**		**Healthy children** ***N*** ** = 19,589**		**COR (CI)**^a^
	>***N***	**Row %**	***N***	**Row %**	
**Locality (Urban/ Rural)**					
Urban	894	9.2	8813	90.8	Urban vs. Rural: 1.3 (1.2–1.4)**
Rural	833	7.2	10,776	92.8	
**Social class**					
Low	576	8.3	6348	91.7	low vs. middle: 1.0 (0.8–1.1)
Middle	615	8.7	6478	91.3	middle vs. high: 1.2 (1.1–1.4)**
High	536	7.3	6763	92.7	low vs. high: 1.1 (1.0–1.3)*
**Geographical Distribution**					
Cities	358	11.0	2906	89.0	cities vs. lower: 1.5 (1.3–1.7)**
Lower Egypt	609	7.7	7312	92.3	lower vs. frontiers: 1.1 (0.9–1.3)
Upper Egypt	592	7.7	7113	92.3	cities. vs upper: 1.5 (1.3–1.7)**
Frontier	168	6.9	2258	93.1	cities vs. frontiers: 1.7 (1.4–2.0)**
**Sex**					
Boys	1067	9.6	10,009	90.4	boys vs. girls: 1.5 (1.4–1.7)**
Girls	660	6.4	9580	93.6	
**Age category**					
1- < 3 years	573	6.8	7805	93.2	3- < 6 vs. 1- < 3: 1.3 (1.2–1.5)**
3- < 6 years	1154	8.9	11,784	91.1	
**Mother age at giving birth**					
< 18	84	9.0	854	91.0	≥ 35 vs. < 18: 1.0 (0.8–1.3)
18 to < 35	1456	7.9	16,920	92.1	< 18 vs. 18- < 35: 1.1 (0.9–1.4)
≥ 35	170	9.0	1728	91.0	≥ 35 vs. 18- < 35: 1.1 (0.9–1.3)
**Mothers Education**					
Illiterate/ Read & write/ Primary/ Prep (1)	741	9.1	7409	90.9	(3) vs. (1): 0.7 (0.6–0.8)**
High School & technical/ above intermediate (2)	741	7.9	8691	92.1	(2) vs. (1): 0.85 (0.8–0.9)**
University or higher (3)	228	6.3	3394	93.7	(3) vs. (2): 0.8 (0.7–0.9)**
**Fathers Education**					
Illiterate/ Read & write/ Primary/ Prep (1)	664	9.3	6465	90.7	(3) vs. (1): 0.6 (0.5–0.7)**
High School & technical/ above intermediate (2)	774	7.8	9093	92.2	(2) vs. (1): 0.8 (0.7–0.9)**
University or higher (3)	202	5.9	3247	94.1	(3) vs. (2): 0.7 (0.6–0.9)**
**Mothers´ work**					
Unemployed (1)	1467	8.0	16,758	92.0	(1) vs. (2): 1.0 (0.9–1.1)
Employed (2)	241	8.1	2735	91.9	
**Presence of mothers or fathers**					
No father at home	87	10.1	775	89.9	No father vs. father at home: 1.3 (1.0–1.6)* No mother vs. mother at home: 2.4 (1.5–4.0)** No mother versus No father at home: 1.9 (1.1–3.2)*
No mother at home	19	17.4	90	82.6	
**Twin child**					
Twins	106	10.9	869	89.1	Twin vs. no twin: 1.4 (1.1–1.7)**
No twins	1621	8.0	18,720	92.0	
Disabled mother					
Mothers with disability	27	16.4	138	83.6	Disabled mother vs. no disability: 2.3 (1.5–3.4)**
No disability	1683	8.0	19,367	92.0	
**Disabled father**					
Father with disability	59	18.5	260	81.5	Disabled father vs. no disability: 2.7 (2.0–3.6)**
No disability	1581	7.9	18,553	92.1	

^*^ = *p*-value significant at < 0.05, ** = *p*-value highly sig at < 0.01, *CI* Confidence Interval, *COR* Crude Odds Ratio

The first variable written in the column is considered as the reference (risky or protective variable)

Concerning the epidemiological factors, children aged 3 – < 6 years were the most likely to be diagnosed with any disability with a prevalence of 8.9% which was significantly 1.3 times higher than the prevalence among the age group 1- < 3 years (COR = 1.3, 95% CI: 1.2–1.5). Boys were one and a half times more likely than girls to be diagnosed with any disability (COR = 1.5, 95% CI: 1.4–1.7).

The age of mothers at giving birth did not show any influence on the occurrence of disabilities. Meanwhile, living without mothers and/or fathers in homes increased the chance of having disabilities by nearly two and half times (COR = 2.4, 95% CI: 1.5–4.0) and 1.3 times respectively (COR = 1.3, 95% CI: 1.0–1.6). Mothers’ work did not seem to affect the odds of having a disability.

Children with mothers or fathers who had higher education were significantly less likely to have any type of disability with the least chance for the mothers and fathers who had a college or greater education level (COR = 0.8, 95% CI: 0.7–0.9 & COR = 0.7, 95% CI: 0.6–0.9 respectively). Children of disabled mothers and/or fathers carried more than two times and nearly three times (COR = 2.3, 95% CI: 1.5–3.4 & COR = 2.7, 95% CI: 2.0–3.6 respectively) the odds to have a disabled child. Whereas being twins increased the odds of having a disability by nearly one and a half times (COR = 1.4, 95% CI: 1.1–1.7).

**Fig. 3:**
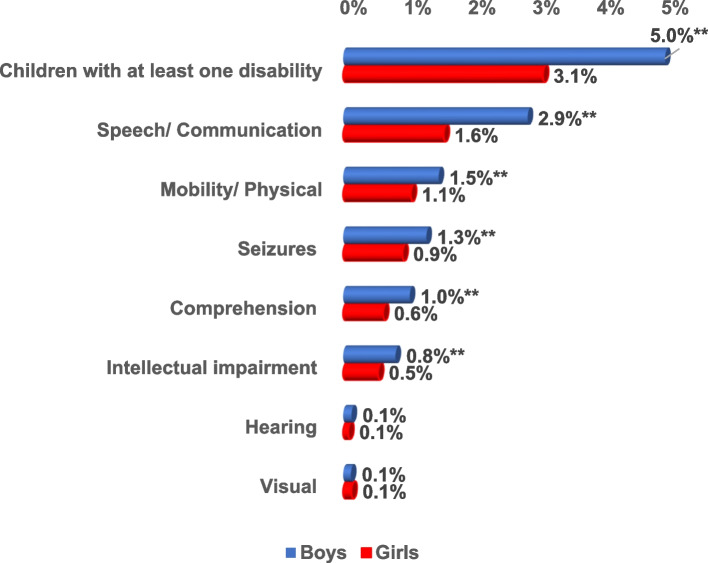
Prevalence of the types of disabilities by sex out of the 21,316 surveyed Egyptian children aged 1– < 6 year. ** = *p*-value highly sig at < 0.01

Among the 21,316 surveyed children, the prevalence of at least one type of disability among boys was significantly higher than that among girls (5.0% and 3.1% respectively). The most prevalent disabilities were speech disability among both males and females (2.9% and 1.6% respectively) followed by mobility/ physical disability (2.9% and 2.2% respectively). Visual disability was seen more among girls (0.3%). Whereas hearing (0.3%), comprehension (2.0%), intellectual impairment (1.6%), and seizures (2.5%) were seen more among boys. This difference in sex distribution was statistically significant except for visual and hearing disabilities (Fig. 3).

**Fig. 4:**
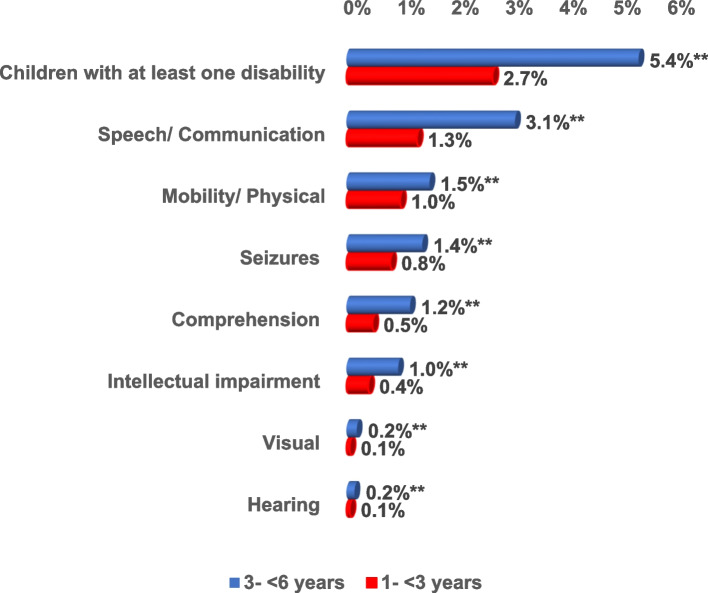
Distribution of the types of disabilities per age group out of the 21,316 surveyed Egyptian children aged 1– < 6 year. ** = *p*-value highly sig at < 0.01

Out of the 21,316 surveyed children, the prevalence of at least one type of disability among children aged 3 – < 6 years was significantly higher than that among children aged 1- < 3 years (5.4% and 2.7% respectively). Speech disability was the most common in children belonging to 1- < 3 years and 3- < 6 years (1.3% and 3.1% respectively) followed by mobility/ physical disability (1.0% and 1.5% respectively). All forms of disabilities were statistically significantly higher among children aged 3- < 6 years than among children aged 1- < 3 years (Fig. 4).

**Fig. 5:**
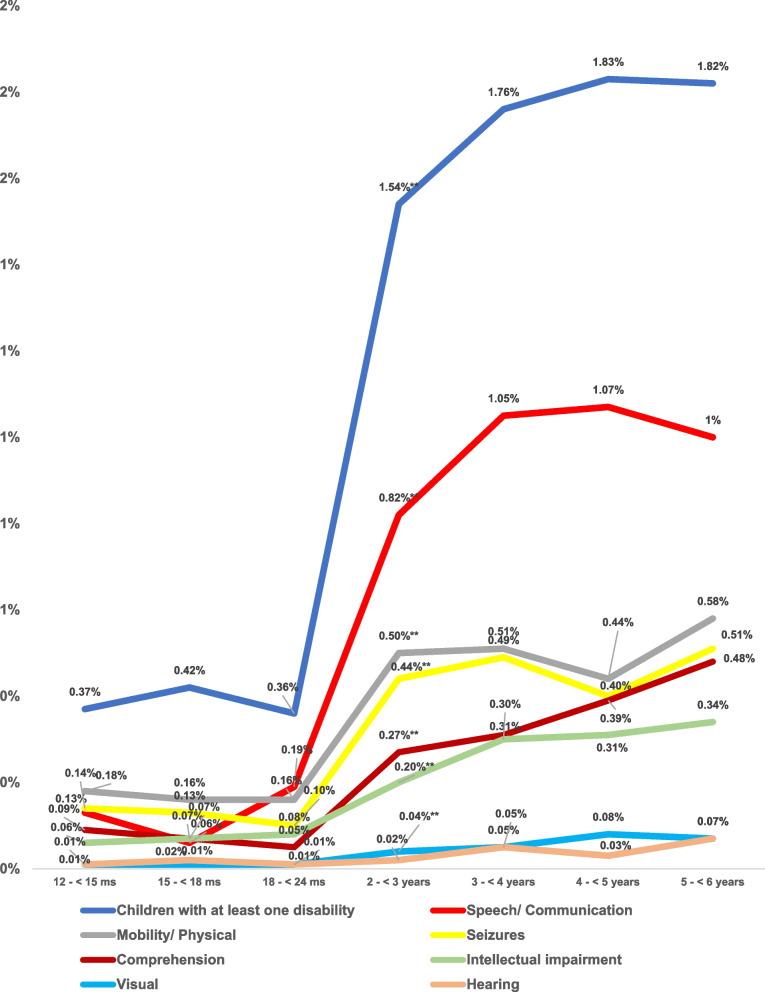
Pattern of distribution of disabilities by age out of the 21,316 surveyed Egyptian children aged 1– < 6 year. ** = p-value highly sig at < 0.01

The pattern of distribution of disabilities by age out of the 21,316 surveyed Egyptian children aged 1– < 6 year was shown in Fig. 5. The prevalence of all the studied disabilities was significantly lower for the age groups below 24 months of age than among children aged more than 2 years except for the visual and hearing disabilities. The prevalence of any form of disabilities especially speech and visual disabilities was highest among children aged 4- < 5 years (1.83%, 1.07 and 0.08% respectively out of the 21,316 surveyed children aged 1- < 6 years). Meanwhile, the prevalence of mobility/ physical, seizures, comprehension, intellectual impairment, and hearing disabilities was highest among children aged 5- < 6 years (0.58%, 0.51%, 0.48%. 0.43% and 0.07% respectively out of the 21,316 surveyed children aged 1- < 6 years). However, insignificant difference could be detected between the two age groups.

Table 3 shows the data of the multivariate logistic regression model for exploring the predictors of disabilities among children aged 1year to < 6 years. The odds of many disabilities was higher among male children by more than one and a half times for hearing,speech, mobility/ physical and comprehension disabilities and intellectual impairment (AOR = 1.6,, 95% CI: 0.8–2.9 AOE = 1.7, 95% CI:1.5–2,AOR = 1.3, 95% CI:1.1–1.3, AOR = 1.5, 95% CI1.2–1.9: & AOR = 1.3, 95% CI:1.1–1.7 respectively). Being an older child was found to have higher odds of having visual disabilities (AOR = 1.4, 95% CI:1.1–1.7). Belonging to the middle social class was associated with a higher odd of having mobility/ physical and comprehension disabilities. Whereas higher paternal education was associated with lower odds of having speech and mobility/ physical disabilities (AOR = 0.7, 95% CI: each 0.6–0.9, 0.5–0.9 respectively), comprehension disability (AOR = 0.5, 95% CI:0.4–0.8), intellectual impairment (AOR = 0.6, 95% CI:0.4–0.9), and seizures (AOR = 0.3, 95% CI:0.1–0.7). Living in Frontiers governorates was associated with higher odds of having both visual and hearing disabilities (AOR = 4.7, 95% CI: 1.6–13.8 & AOR = 14.5, 95% CI:3.1–69 respectively). Living in lower Egypt decreases the chance to have both mobility/ physical disabilities (AOR = 0.7, 95% CI:0.5–0.9) and comprehension disabilities (AOR = 0.6, 95% CI:0.4–0.9) compared to living in cities. Whereas living in upper Egypt increases the chance to have only intellectual impairment disability by nearly one and a half (AOR = 1.5, 95% CI:1.5–2.2) than living in cities. Living in urban communities was more protective for mobility, comprehension, and intellectual impairment disabilities than living in rural communities (AOR = 0.8,, 95% CI:0.7-0.09 AOR = 0.7, 95% CI:0.5–0.9 & 0.7, 95% CI: 0.5–0.9 respectively).

**Table 3:** Multiple Logistic regression model for prediction of diagnosis. (Each type of disability vs healthy without this type of disability)

Parameters	Visual		Hearing		Speech/ Communication		Mobility/ Physical		Comprehension		Intellectual impairment		Seizures	
	**AOR**	**CI**	**AOR**	**CI**	**AOR**	**CI**	**AOR**	**CI**	**AOR**	**CI**	**AOR**	**CI**	**AOR**	**CI**
Age	1.4**	1.1	1.1	0.9	1.2**	1.1	1.0	0.9	1.2**	1.1	1.2**	1.1	1.0	0.9
		1.7		1.4		1.2		1.1		1.3		1.3		1.1
Sex (male)	0.8	0.5	1.6	0.8	1.7**	1.5	1.3**	1.1	1.5**	1.2	1.3*	1.1	1.0	0.7
		1.4		2.9		2.0		1.5		1.9		1.7		1.5
Locality (urban- rural) urban is the base	1.8	0.9	1.5	0.7	0.8**	0.7	0.8	0.7	0.7**	0.5	0.7**	0.5	1.3	0.6
		3.7		3.0		0.9		1.0		0.9		0.9		2.4
Social level (High to low)	1.0	0.5	2.3*	1.0	1.0	0.8	0.9	0.7	1.3	0.9	1.2	0.9	1.1	0.7
		1.8		5.2		1.2		1.2		1.7		1.7		1.6
Social level (Middle to high)	0.6	0.3	1.8	0.8	1.2	0.9	1.6**	1.3	1.7**	1.3	1.3	0.9	0.9	0.3
		1.2		4.3		1.4		2.0		2.2		1.7		1.3
Geographical (Frontiers to cities)	4.7**	1.6	14.5**	3.1	0.8	0.6	1.2	0.9	1.0	0.7	1.5	0.9	0.6	0.2
		13.8		69.0		1.1		1.7		1.5		2.3		1.5
Geographical (Lower to cities)	0.8	0.3	1.6	0.3	0.8	0.7	0.7*	0.5	0.6**	0.4	1.1	0.7	0.9	0.5
		2.4		8.4		1.1		0.9		0.9		1.6		1.8
Geographical (Upper to cities)	1.2	0.4	2.6	0.5	0.8	0.7	1.0	0.7	1.0	0.7	1.5*	1.0	1.9	0.9
		3.6		13.0		1.0		1.3		1.4		2.2		3.7
Maternal Education (University and above to less education)	0.9	0.4	1.5	0.5	1.0	0.8	1.0	0.7	1.3	0.9	1.6**	1.1	1.5	0.8
		2.6		4.6		1.3		1.3		1.8		2.4		2.7
Paternal Education (University and above to less education)	1.0	0.4	0.7	0.2	0.7**	0.6	0.7*	0.5	0.5**	0.4	0.6**	0.4	0.3**	0.1
		2.6		2.3		0.9		0.9		0.8		0.9		0.7
Twins	1.8	0.7	1.0	0.2	1.1	0.8	0.9	0.6	0.9	0.6	1.0	0.6	0.4	0.1
		4.6		4.2		1.5		1.3		1.4		1.7		1.3
Disabled mother	2.5	0.5	10.8**	2.5	1.4	0.8	2.0*	1.0	0.2	0.03	0.7	0.2	1.7	0.4
		12.1		47.6		2.6		4.1		1.7		2.8		7.5
Disabled father	5.9**	2.1	0.0	0.0	1.3	0.8	1.3	0.7	1.4	0.7	0.8	0.3	1.5	0.4
		16.3		-		2.1		2.3		2.8		2.2		4.9
Mother have any health problem during pregnancy	3.1**	1.4	2.5	0.9	1.7**	1.4	1.7**	1.3	1.7**	1.2	2.3**	1.6	1.3	0.7
		6.8		6.7		2.1		2.3		2.4		3.3		2.5
Difficult labor	0.5	0.2	1.4	0.6	1.0	0.9	1.0	0.8	1.2	0.9	1.0	0.7	1.3	0.8
		1.2		3.3		1.2		1.3		1.7		1.4		2.0
Preterm delivery (child born less than 7 months)	3.7*	1.1	1.6	0.2	0.9	0.6	1.2	0.7	1.3	0.7	1.0	0.5	1.0	0.6
		12.1		16.0		1.5		2.0		2.4		2.2		2.3
Baby’s weight less than 2.5 kg at birth	0.9	0.3	0.2	0.02	1.6**	1.3	2.4**	1.8	2.0**	1.4	2.0**	1.4	2.2*	1.2
		2.5		2.4		2.1		3.1		2.8		3.0		3.9
Child suffer from jaundice after birth	1.2	0.6	0.6	0.3	1.2*	1.0	1.0	0.8	0.9	0.7	0.9	0.7	0.9	0.6
		2.3		1.5		1.4		1.2		1.1		1.2		1.3
Child suffer from bluish discoloration after birth (cyanosis)	1.2	0.3	1.6	0.3	1.4	0.9	1.4	0.9	1.0	0.5	1.0	0.5	4.7**	2.2
		5.3		9.5		2.1		2.2		1.8		2.0		10.3
Child suffer from convulsions after birth	1.5	0.4	8.1**	2.2	2.5**	1.8	3.4**	2.3	3.6**	2.3	4.2**	2.5	2.6 × 10^11^	0.0
		5.6		30.5		3.5		5.0		5.7		6.9		-
Child kept in an incubator for more than two days	3.7**	1.7	1.2	0.3	1.6**	1.3	1.8**	1.4	2.1**	1.5	1.6*	1.1	3.0**	1.8
		7.9		4.0		2.0		2.4		2.8		2.3		5.0
Constant	0.0**		0.0**		0.0**		0.0**		0.0**		0.0**		0.0**	
Model fit (Cox & Snell R Square)	0.524		0.345		0.678		0.414		0.959		0.724		0.263	
Percent correctly predicted (Hosmer and Lemeshow Test)	0.3%		0.3%		1.6%		1.3%		1.1%		0.7%		13.9%	

Variable(s) entered in model 1: age, sex, locality, social class, geographical distribution, twins, mother education, father education, mother disability, father disability, mother problem during pregnancy, difficult labor, child born less than 7 m, baby weight less than 2.5 kg at birth, child suffer from jaundice after birth, child suffer from bluish discoloration after birth, child suffer from convulsions after birth, child kept in incubator for more than two days

^*^ = *p*-value significant at < 0.05, ** = *p*-value highly sig at < 0.01, *AOR* Adjusted Odds Ratio, *CI* Confidence Interval

The strong predictors for all disabilities were as follows: neonatal history of convulsions, being kept in an incubator for more than two days, and if mothers had a history of any health problem during pregnancy. Whereas low birth weight acted as a risk factor for speech, mobility/ physical, and comprehension disabilities, intellectual impairment, and seizures with odds ratios for disability from one and a half for speech disability (AOR = 1.6, 95% CI:1.3–2.1) to more than twice for mobility/ physical disability (AOR = 2.4, 95% CI:1.8–3.1). Neonatal history of cyanosis after birth was a predictor for visual (AOR = 1.2, 95% CI:0.3–5.3), hearing (AOR = 1.7, 95% CI:0.3–9.5), speech and mobility/ physical disabilities (AOR = 1.4, 95% each CI:0.9–2.1,0.9–2.2 respectively), and seizures (AOR = 4.7, 95% CI:2.2–10.3). In addition, preterm delivery carried significantly four times the odds for visual disability (AOR = 3.7, 95% CI: 1.1–12.1). Children of disabled mothers and fathers were more likely to have visual, speech & mobility/ physical disabilities and seizures. Having a disabled mother was a strong predictor for hearing disability (AOR = 10.8, 95% CI: 2.5–47.6) and physical mobility (AOR = 2.0, 95% CI: 1.0–4.1). Whereas having a disabled father was a predictor for visual disability (AOR = 5.9, 95% CI: 2.1–16.3). Neither the history of difficult labor nor that of twins was a significant predictor for any disabilities.

## Discussion

More than one billion people in the world suffer from some form of disability [39], 150 million of which are children [40]. Given the emphasis of the global developmental goals on reducing childhood mortality, most countries have given less priority to tackling childhood disability [41].

This study was conducted to assess the national prevalence of disabilities among 21,316 Egyptian children aged 1 to < 6 years utilizing the WHO Ten questions screening tool (TQS).

In the current study, the prevalence of disability among children aged 1 – < 6 years was found to be 8.1% (at least one type of disability). The detected prevalence is higher than those reported in other countries. The international prevalence of child disability was found to be 5% worldwide [42] and 7.3% in the UK [43].

Data from Egypt and Arab countries is relatively scarce and old [11, 44]. According to the National Survey of Children’s Health (NSCH) from 2016 to 2018 disability ranged from 4.9% to 14% [45]. The latest available survey from Egypt on screening for disabilities among preschool children was published in 2005. It reported high variability in rate (0.7% -8.8%) and was conducted in a limited number of governorates [11]. A minimal decline was observed globally over the same period [2] but the percentage is still high. This may be explained by the development of new research tools and the improvement of parent awareness.

In the current study, different types of disability were investigated including speech, communication, mobility/ physical, seizures, comprehension, and intellectual impairment. The highest prevalence was for speech/ communication (4.4%) and the least was for hearing (0.2%).

A speech disorder is a common developmental difficulty in childhood. It may be due to another condition such as autism, hearing impairment, general developmental difficulties, behavioral or emotional difficulties, or neurological impairment), or it may be considered primary when it cannot be accounted for by any other condition [46, 47].

Our findings are consistent with many other studies [48–50] and in contrast with other studies [51]. Like other studies, speech and language delay accounts for a high percentage of child disabilities (up to 27% in children > 3 years) [52] and ranged between 10- 18% in children 1–6 years in India [53, 54] the prevalence was 2.5% by other authors [55]. This wide percentage range in speech disorders is at least in part due to the different tools used in surveying. This illustrates how changes to disability definitions and tool sets within surveys can affect prevalence estimates.

The present study reported an association between age and disability prevalence. The prevalence of at least one type of disability among children aged 3 – < 6 years was 5.4% which was 1.3 times higher than the prevalence among the age group 1- < 3 years (2.7%).

This finding could be explained by the fact that most disabilities do not manifest early. Some become progressively more activity-limiting as the child gets older [43]. In addition, older children (3- < 6 years) start to go out of their homes where they may be subjected to brain injury due to motor vehicle crashes, falls, sports injuries, and physical abuse. Brain injury is an important cause of disability [56]. Another explanation, children at this age are admitted to kindergartens, where they interact with peers and child care educators who can easily observe cognitive disability or any other potential disability [57].

Early pediatric diagnosis should become a routine practice of monitoring children from infancy to school age [58]. Moreover, early detection of disability may prevent the development of other disabilities or developmental delays [33].

Among the surveyed children, the prevalence of disability was 5.5% among boys and 3.2% among girls. This is consistent with many epidemiological studies that report a higher prevalence of language delay, communication disorders, and speech disorders in males than in females [48, 59, 60]. Some authors explained this by the fact that the normal process of communication and language skills development is faster and more advanced in girls compared to boys [61]. Other authors owe the cause to the effect of testosterone on language-related brain areas, as well as functional communication and language skills [62, 63].

Although hearing loss is a well-documented cause of speech delay [64], the hearing was the least prevalent type of disability in our study (0.3%). A few years ago, Egypt adopted a national screening survey for newborns for early detection and early interference with hearing disability which is expected to have a positive impact on communication skills later [65].

The causes of disabilities probably interact with each other, and it is difficult to analyze each cause separately even starts early in life with developmental delays [66, 67]. Our study found that belonging to the middle social class was a major predictor for mobility/ physical and comprehension disabilities (associated with nearly twice the risk for these disabilities). We also found a significant difference in all disabilities, especially intellectual disabilities between the middle and high social levels. This was consistent with an Egyptian study comparing the cognitive, motor, and communication scores of middle social-class Egyptian infants and toddlers with that of the reference norms [68]. On Bayley Scales of Infant and Toddler Development-third edition (Bayley III), the mean values of all assessed developmental domains were below that of the American norms which could contribute to the higher prevalence of disabilities especially the intellectual ones [68].

By analyzing the sociodemographic factors, we found that the risk of having at least one disability in cities was significantly higher than in frontier, upper, and lower Egypt governorates with the prevalence of 11%, 6.9%, 7.7%, and 7.7% respectively. Generally, the risk to have at least one type of disability was 1.3 times higher in urban compared to rural communities (prevalence 9.2% vs. 7.2%) and among middle and low compared to a high social class (prevalence 8.7% and 8.3% respectively vs. 7.3%). Data from other countries suggests that the proportion of people with disabilities living under the poverty line is higher than that of people without disabilities – in some countries, twice as high [69].

However, the situation differs when studying individual disabilities, in rural areas, people with disabilities have a tendency to face more challenges than their counterparts in urban areas for some of the studied disabilities that were linked to intellectual impairment and comprehension. Disabled people are less likely to attend school, less likely to be employed, and less likely to be served by a skilled health worker. Consequently, they are often left behind in rural development interventions. In other developed and developing countries, still, the disability rates are higher in more rural than in urban areas for some disabilities [70, 71]. This finding highlights the importance of community-based interventions that proved to be effective in raising rural population awareness mainly for rural women [72, 73].

Generally, social and environmental factors are interrelated, and all factors influence each other including parental education and the social class of the family. This was established in our findings as we reported that children with mothers or fathers who had higher education were significantly less likely to have any type of disability with the least risk for the mothers and fathers who had a college or greater education level. Mothers’ work did not seem to affect the risk of having a disability. Childhood disability often requires family adjustments in terms of both time and money that may have lasting psychological and economic consequences for all family members. Many parents, primarily mothers, choose to stay at home while their children are young, reentering the workforce sometime after their youngest child enters elementary school. However, for families with children who have disabilities, the decision of one parent not to work may be more of a necessity than a choice. As important as this decision is, its economic aspect is still overlooked [74].

Parents’ education also has an impact on children’s health. Higher paternal education was found to decrease the chance of having speech and mobility/ physical disabilities by 30%, intellectual impairment by 40%, and seizures by 70%. Maternal education was found to have an even greater role [75–78]. Others have studied the impact of parent education on child health [79, 80].

Several authors demonstrated the effect of low parental education in speech development. Importantly, maternal nutrition education severs as a protective factor for children's health [81–83].

Living without mothers and/or fathers in homes increased the chance of having disabilities by 2.5 and 1.3-fold respectively. Meanwhile, the age of mothers at the time of birth did not show any influence on the occurrence of disabilities. Earlier research has demonstrated that raising a child with a disability can strongly impact families, leading to higher divorce rates, and poorer well-being for caregivers [74, 84].

Maternal health during the antenatal period is a critical risk factor for child health [85]. The survivors of complicated pregnancy and neonatal incubator admission often suffer from neuro-developmental consequences. This is consistent with our findings as we reported that a neonatal history of convulsions or being kept in an incubator for more than two days and if mothers had a history of any health problem during pregnancy is a strong predictor for all disabilities.

It is well-studied that children born preterm are at greater risk of visual impairment than their peers. Furthermore, low birth weight, low Apgar score, and higher birth order were found to be risk factors for disabilities such as speech [86–88]. Other researchers didn’t find a significant relation [83]. We documented that low-birth-weight acts as a risk predictor of speech, mobility/ physical, and comprehension disabilities, intellectual impairment, and seizure.

Many Egyptian studies reported that promoting children’s psychological and social development and reducing disabilities are highly linked to the provision of adequate maternal health care during pregnancy, childbirth and during early childhood [89–91]. Even later in life, supporting children's growth and development is required at school age through nutritional supplements and education [90, 92].

## Strengths and limitations of the study

One of the strengths of this study is the large sample size. Moreover, the household survey technique and the tools used are designed to make significant comparisons of disability prevalence between countries. The respondents were enrolled using the probability sampling technique to enhance the representativeness of the study.

The study is in alignment with the United Nations Convention on the Rights of Persons with Disabilities to be served and for not leaving any child behind. Moreover, it is in alignment with Sustainable Development Goal 10. The data provided by this study are considered the base for building strategies to support the social development and education of children with difficulties.

This study has some limitations: the first one was that the WHO TQS has not been validated for the Egyptian study population. As this research is based on a cross-sectional population screening test, the study only focused on the risk factors that carry associations of each type of disability but could not assess the role of the environmental or the genetic factors as a contributing factor to some of the studied types of disability. Moreover, although disability is well known to have serious impact on the quality of life, this could be more evident in the Arab countries as rehabilitation centres are usually paid for out of patients’ pockets. However, this study could not assess such aspects.

## Conclusion

We reported the overall disability prevalence which ranged from 4.4% (speech) to 0.2% (hearing). The coincidence of many disabilities was higher among male children and was significantly higher for speech, mobility/ physical and comprehension disabilities, and intellectual impairment. Children aged 3 – < 6 years were the most likely to be diagnosed with any disability. The strong predictors for all disabilities were neonatal history of convulsions, being kept in an incubator for more than two days, and if mothers had a history of any health problems during pregnancy. Low birth weight was found to be a risk predictor for speech, mobility/ physical and comprehension disabilities, intellectual impairment, and seizures. Children of disabled mothers and fathers were at higher risk of having visual, speech & mobility/ physical disabilities and seizures. Disabled mother was a significantly strong predictor for hearing disability.

### Supplementary Information

**Additional file 1:** **S Table 1.** List of the expected and targeted children according to the governorates, locality and sociodemographic status for screening of disability among children aged 1-6 years.

**Additional file 2: S Table 2.** All data disability less than 6.

## Data Availability

The datasets used and/or analyzed for the current study are anonymous and are fully available without restriction as S Table 2 Raw data (XLSX).

## Abbreviations

MOHP: Ministry of Health and Population
SPSS: Statistical Package for Social Sciences
TQS: Ten questions screening tool
UK: United Kingdom
UNCRPD: Convention on the Rights of Persons with Disabilities
UN: United Nations
UNFPA: United Nations Population Fund, formerly the United Nations Fund for Population Activities
WG: Washington Group on Disability Statistics
WHO: World Health Organization
