# Supplementary material for: Prevalence and risk factors of disabilities among Egyptian preschool children: a community-based population study
Source: BMC Psychiatry. 2023 Sep 21;23:689. doi: 10.1186/s12888-023-05171-3 (PMC10514965; doi:10.1186/s12888-023-05171-3)
Supplement: Supplementary file 1 — Additional file 1: S Table 1. List of the expected and targeted children according to the governorates, locality and sociodemographic status for screening of disability among children aged 1-6 years. [file 12888_2023_5171_MOESM1_ESM.docx]

| serial | **Gov.** | **classification according to HDI** | **Kesm/Markaz** | **Urban** | | |  | **Rural** | | | Total | |
| --- | --- | --- | --- | --- | --- | --- | --- | --- | --- | --- | --- | --- |
|  |  |  |  | **Shiakha (English)** | **(HH)Expected children** | **Targeted child** | **Local Unit ( (English )** | **Village (English )** | **(HH)Expected children** | **Targeted child** | **(HH)Expected children** | **Targeted child** |
| **1** | **Cairo** | **High** | **Al Nozhah** | **Al Hicksit** | **1350** | **1257** |  |  |  |  | 1350 | **1257** |
|  |  | **middle** | **Al Saiedah Zainab** | **Alkabsh** | **1350** | **1250** |  |  |  |  | 1350 | **1250** |
|  |  | **low** | **el Sharabia** | **Al Amiria** | **1350** | **1252** |  |  |  |  | 1350 | **1252** |
| **2** | **Dakhlya** | **High** | **Al Senbelawin** | **Al Sinblaween city** | **317** | **306** | **Kafr Alruwk** | **Alshalaa** | **905** | 848 | 1222 | **1154** |
|  |  | **middle** | **Markaz of MietSalsil** | **Mit salsil city** | **317** | **296** | **Alatihad** | **AlJafara** | **905** | 843 | 1222 | **1139** |
|  |  | **low** | **Al Gamaliah** | **Almataria city** | **317** | **334** | **Alsafra** | **Al Dahear** | **905** | 836 | 1222 | **1170** |
| **3** | **Gharbia** | **High** | **Kafr el Zaiat** | **KafrElZaiat** | **260** | **247** | **Kafour Belshay** | **Qasta** | **680** | 640 | 940 | **887** |
|  |  | **middle** | **Samanood City** | **Samanood** | **260** | **246** | **Ziyad’s locality** | **Munshat Nzif** | **680** | 647 | 940 | **893** |
|  |  | **Low** | **Markazof Qutour** | **Qutour** | **260** | **254** | **Kotour** | **Khabata** | **680** | 586 | 940 | **840** |
| **4** | **Fayoum** | **High** | **Al Fayoum City** | **Alqism rabie** | **145** | **155** | **Dacia** | **Al Sunbat** | **580** | 589 | 725 | **755** |
|  |  | **middle** | **Markazof Senoures** | **Senoures** | **145** | **140** | **Terrsa** | **Alzawia El Khadra** | **580** | 518 | 725 | **658** |
|  |  | **Low** | **Markazof Tamiaha** | **Tamiaha** | **145** | **144** | **Sarsna** | **Kafr Omira** | **580** | 552 | 725 | **696** |
| **5** | **Assuit** | **High** | **Assyout city** | **Alwalidia Alwustania** | **235** | **240** | **Bani Hussein** | **Musriea** | **795** | 868 | 1030 | **1108** |
|  |  | **middle** | **Al Kousiah City** | **Al Kousiah City** | **235** | **276** | **Mir** | **Bani Hilal** | **795** | 987 | 1030 | **1263** |
|  |  | **Low** | **Al Ghanaiem** | **Al Ghanaiem** | **235** | **281** | **Alazayiza** | **Al Amri** | **795** | 740 | 1030 | **1021** |
| **6** | **Aswan** | **High** | **Nasr Al Nouba City** | **Nasr Al Nouba City** | **280** | **279** | **Korta** | **Garf Hussein** | **410** | 418 | 690 | **697** |
|  |  | **middle** | **Edfo City** | **Al-Busaliya Bahri** | **280** | **302** | **Alramad Albahry** | **Adfu Quabli** | **410** | 374 | 690 | **676** |
|  |  | **Low** | **MarkazKoomOmbo** | **KoomOmbo** | **280** | **275** | **Al Abbasia** | **Sabaa Quabli** | **410** | 378 | 690 | **6533** |
| **7** | **Damietta** | **High** | **Farskor** | **AlRawda** | **275** | **271** | **Hajaja Village** | **Hajaja Village** | **380** | 376 | 655 | **647** |
|  |  | **middle** | **Al zarqaa** | **Alsarw** | **275** | **275** | **Sharmsah** | **Kafr Toqaa** | **380** | 378 | 655 | **653** |
|  |  | **Low** | **Kafrsaad** | **Kafer Albatiykh** | **275** | **271** | **Kafr Saad Country** | **Nawasiriya village** | **380** | 410 | 655 | **681** |
| **8** | **Marsa Matrouh** | **High** | **Marsa Matrouh** | **MarsaMatrouh**  **(Alsanusia & Kilo 4)** | **470** | **471** | **Alkasr** | **Alkasr** | **220** | 231 | 690 | **702** |
|  |  | **middle** | **Al Hamam City** | **Al Hamam City** | **470** | **462** | **Alsalam** | **Alsalam** | **220** | 225 | 690 | **687** |
|  |  | **Low** | **KismSaiedy Barany** | **AlNajyla** | **470** | **453** | **Almathany** | **Almathany** | **220** | 211 | 690 | **664** |
| **Total** | | | | | **9996** | **9737** |  | | **11910** | **11655** | **21906** | **21392** |

S Table-1: List of the expected and targeted children according to the governorates, locality and sociodemographic status for screening of disability among children aged 1-6 years
